# Os Cuboideum Secundarium: A Rare Accessory Ossicle in the Foot

**DOI:** 10.5334/jbsr.3043

**Published:** 2023-03-13

**Authors:** Arne Vermeulen, Nirav Gupta, Seema Döring

**Affiliations:** 1 UZ Brussel, BE; 2 LTM Medical College and General Hospital, IN

**Keywords:** Os Cuboideum Secundarium, Accessory Ossicle, Ankle distortion, Bone Marrow Edema, Fracture

## Abstract

**Teaching Point:** Knowledge of the accessory ossicles in the foot and the possible disorders of these ossicles are important to prevent misdiagnosis and mistreatment.

## Case History

**Figure 1:**
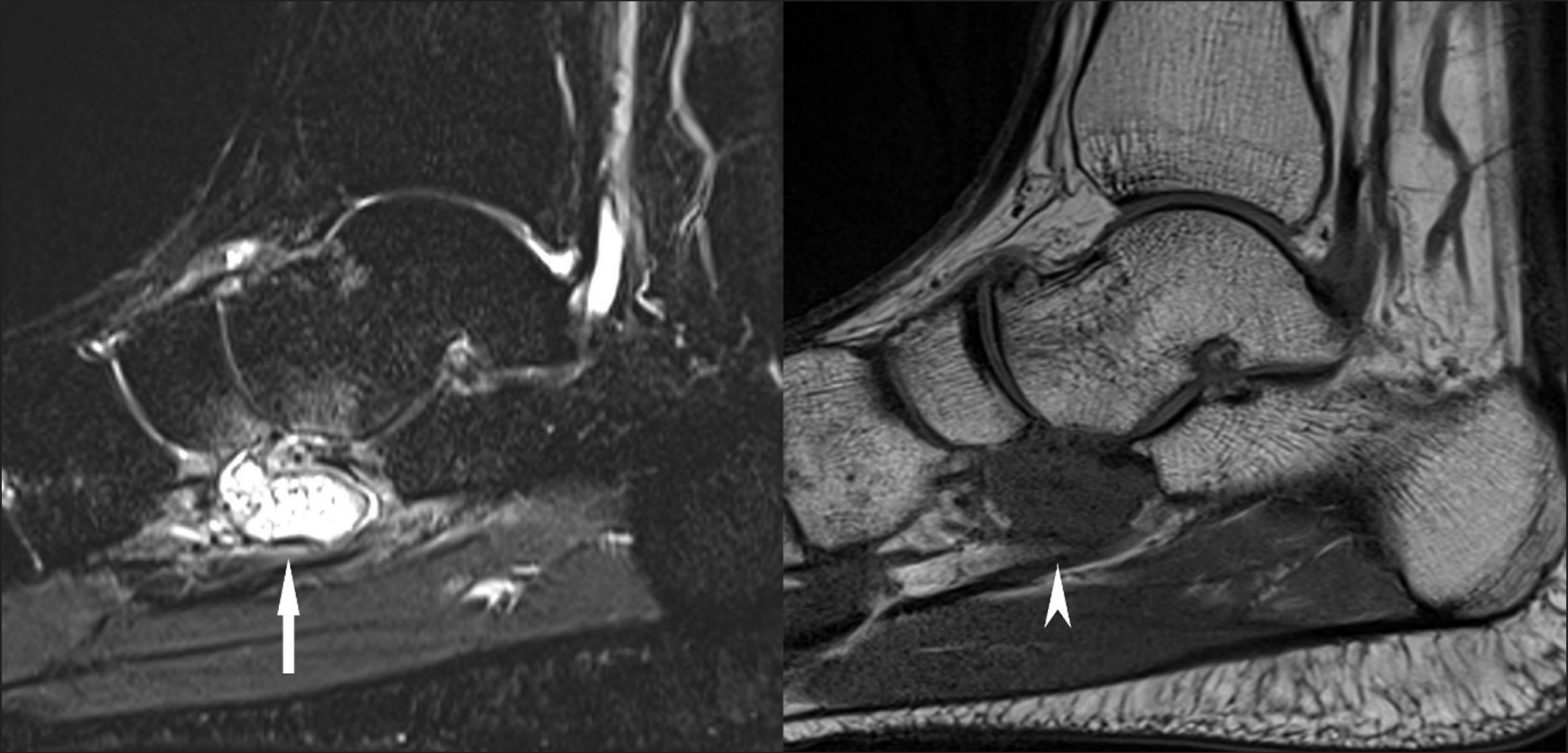


A 32-year-old male presented with persistent right ankle pain four weeks after ankle distortion without clear signs of swelling. The referring clinician suspected ligament injury. To evaluate the ankle injury, a non-contrast magnetic resonance imaging (MRI) examination was performed. The MRI demonstrated a mass adjacent to the calcaneocuboid articulation and inferior to the talonavicular articulation. The mass had very high signal intensity on STIR imaging (Figure 1, arrow) and low signal intensity on T1-weighted images (Figure 1, arrowhead). There was bone marrow edema in the plantar aspect of the talonavicular articulation. There was no edema in the surrounding soft tissues nor were there injuries to the ankle ligaments or other abnormalities.

**Figure 2:**
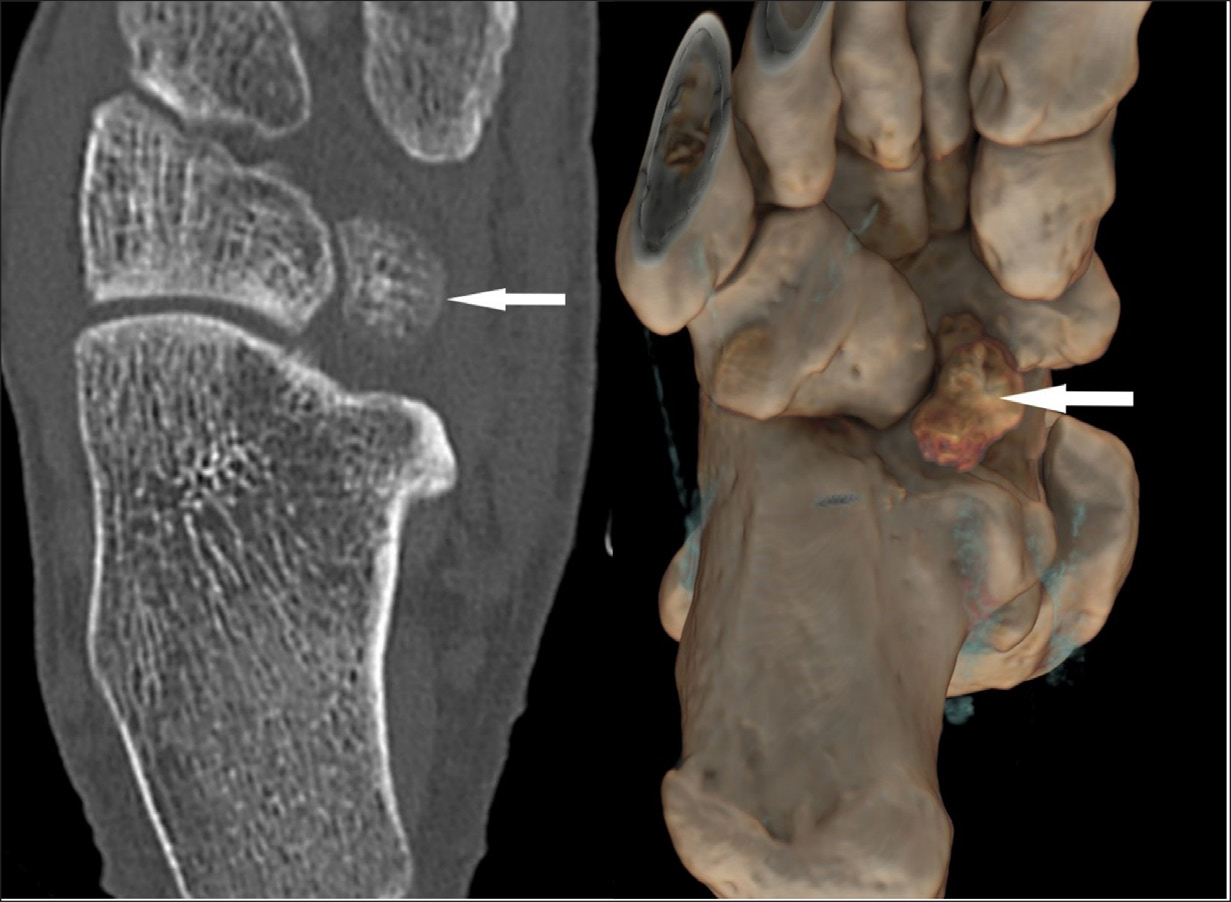


To clarify the findings on MRI, an additional computed tomography (CT) examination was performed. This demonstrated a 23 × 15 × 12 mm ossific density medial to the inferior margin of the cuboid and anterior to the inferior margin of the calcaneus (Figure 2, arrows) consistent with a very rare accessory ossicle called the os cuboideum secundarium.

Despite the extensive bone marrow edema seen in the os cuboideum secundarium on MRI there was no fracture evident on CT. As there were no other abnormalities seen on MRI and CT imaging, bone marrow edema in the accessory ossicle and the plantar aspect of the talonavicular articulation are the most likely cause of the ankle pain. A stress reaction of the ossicle with the talonavicular articulation after trauma was the favored diagnosis.

## Comments

Accessory ossicles are normal variants in bone development which originate from unfused secondary ossification centers adjacent to the main bony mass [1]. The foot is the most common site of accessory skeletal elements and as many as 24 accessory ossicles of the foot have been described [1]. The os cuboideum secundarium is a very rare accessory ossicle, of which the exact prevalence is unknown. However there have been at least three case reports which documented pain associated with this accessory ossicle [2,3,4]. The ossicle is located adjacent to the cuboid and calcaneus. Accessory ossicles are usually asymptomatic and incidental findings but can become painful due to multiple reasons such as trauma, of which fractures (acute or stress) and dislocations are the most common [1] or from exogenous pressure of weightbearing [3]. In our patient the pain started similarly to a case found in the literature following a twisting injury of the ankle [2]. The bone marrow edema pattern could be explained by a plantar flexion injury that occurred at the Chopart joint which caused a nutcracker-like motion that happened on the ossicle between the talonavicular articulation. The persistence of pain as well as the more pronounced bone marrow edema in the ossicle as compared to the talonavicular articulation suggests repetitive stress after the initial injury.
